# Prediction of Clinical Outcome for All Stages and Multiple Cell Types of Non-small Cell Lung Cancer in Five Countries Using Lung Cancer Prognostic Index^☆^

**DOI:** 10.1016/j.ebiom.2014.10.012

**Published:** 2014-10-25

**Authors:** Tiehua Chen, Luming Chen

**Affiliations:** 4014S 1000 E, Salt Lake City, UT 84124, United States

**Keywords:** Non-small cell lung cancer, Gene expression profile, Overall survival, Recurrence free survival, Prognosis, Meta-analysis

## Abstract

Lung cancer is a commonly diagnosed cancer. In this era of personalized medicine, genetic predictive models are becoming increasingly important. However, many current predictive models fail verification tests due to small sample sizes and institutional biases. We collected 17 gene expression datasets from public databases to generate our largest training and testing cohorts. After successfully eliminating institutional variations and merging multiple datasets, we generated a training cohort of 1073 and a testing cohort of 659. Using Siggenes, univariate and multivariate analyses, we identified seven gene signatures, and combined them with the clinical parameter age and stage to design the lung cancer prognostic index (LCPI). Using LCPI, we could differentiate lung cancer patients into three risk groups and predict patient survival probabilities at 10 and 15 year post-surgical resection. We extensively verified the predictive ability of LCPI for overall and recurrence free survival using 6 other datasets from five different countries.

## Highlights

• We collected 17 gene expression datasets of non-small cell lung cancer from public databases to generate a training cohort of 1073 and a testing cohort of 659 with institutional variations successfully eliminated.
• We identified seven gene signatures, and combined them with the clinical parameters age and stage, we could differentiate patients into three risk groups and predict patient survival probabilities at 10 and 15 years post-surgical resection, which were extensively verified using 6 other datasets from five different countries.

## 1 Introduction

Lung cancer is a leading cause of death. In 2008, about 12.7 million cases and 7.6 million deaths were reported worldwide (Jemal et al., 2011). Non-small-cell lung cancer (NSCLC) accounts for 85% of all cases of lung cancer, and includes adenocarcinoma (ADC), squamous cell carcinoma (SCC) and large cell carcinoma (LC). Currently, surgical resection is a common procedure for patients with stage I, stage II, and certain subsets of stage IIIA NSCLC (Ramalingam et al., 2011). For patients with stage II, stage IIIA, and select stage IB, adjuvant cisplatin-based chemotherapy (ACT) after surgical resection is the standard of care (Patel and Wakelee, 2011a). However, the effectiveness of using ACT to increase patient survival time remains debatable. In the era of personalized medicine, predictive markers can play a crucial role in helping clinicians to separate patients that may benefit from post-surgical treatments and patients that can be spared the burden of overtreatment.

Gene expression profiles (GEP) are valuable sources of patient data. Since the first publications of GEP for lung cancer in 2001 (Bhattacharjee et al., 2001), many studies have proposed predictive models to estimate patient survival time. These models ranged from a single gene to hundreds of genes (Bild et al., 2006, Takeuchi et al., 2006, Gruber et al., 2006, Raponi et al., 2006, Director's Challenge Consortium for the Molecular Classification of Lung, A., et al., 2008, Lee et al., 2008, Kuner et al., 2009, Lu et al., 2010, Zhu et al., 2010, Hou et al., 2010, Sanchez-Palencia et al., 2011, Xie et al., 2011, Okayama et al., 2012, Botling et al., 2013, Rousseaux et al., 2013, Sato et al., 2013, Tang et al., 2013). Models based on the expression of hundreds of genes is economically impractical in the clinic, and models based on fewer genes have not been verified in different testing cohorts due to small sample size and the variations inherent in data collected from a single institution. Additionally, some authors have truncated data collected over 10 or more years to only 5 years, introducing error in survival predictions and contributing to difficulty in verification. As such, we hypothesize that NSCLC survival time is a quantitative and predictable trait. We have generated a more reliable model by combining multiple datasets obtained from different institutions and different countries to increase the sample size and mitigate the error introduced by institutional biases. We collected 17 publically available NSCLC datasets (Table 1), standardized 11 of them by removing batch effects, and then combined them to form a training cohort of 1073 and a testing cohort of 659 patients, which are the largest two GEP datasets of NSCLC in the world. In doing so, we demonstrated how large datasets can be generated, normalized, and analyzed by pooling resources from multiple investigators and provided a formula for converting gene expression datasets from two-channel to single-channel data.

Additionally, multiple studies indicated that gene expression data combined with clinical parameters can improve the predictive capacity of lung cancer survival models (Director's Challenge Consortium for the Molecular Classification of Lung, A., et al., 2008, Lee et al., 2008). When we analyzed the training cohort, we not only identified seven gene signatures as independent predictive markers, but also found age and stage to be supplementary independent predictors. We designed the lung cancer prognostic index (LCPI) as a predictive score that accounts for the seven biomarkers as well as age and stage, with lower LCPI scores corresponding to higher survival probabilities. Here, we show that we were able to separate the patient populations in the training and testing cohort into three distinct risk groups using the LCPI model. We used 6 other publically available NSCLC datasets as additional testing cohorts for extensive verification and showed that the LCPI model was able to predict patient survival regardless of lung cancer stage, type or country of origin.

## 2 Methods

### 2.1 GEP Data Collection and Grouping

We collected 17 publically available GEP datasets (n = 2738) with clinical parameters from the Gene Expression Omnibus and the National Cancer Institute (GSE26939 (Wilkerson et al., 2012) added breast cancer cells as reference was excluded from our studies). As we needed both the GEP data as well as the corresponding clinical parameters, any dataset that did not release or contain either type of data was excluded from our study. The gene expression data was obtained from tumor tissue after surgical resection, and thus we limited our analysis to patients for whom surgical resection is a viable option. Although the analysis is not shown in this paper, we did explore the effect of prior grouping variables. Most of the data in the 17 studies have similar age range, similar gender distribution, and similar death ratios. As a result of the parameters of the original studies, none of the patients receive preoperative chemotherapy. There were a total of 230 control samples. According to the power calculations, to attain 90% power with a significance level of 0.05 and effect size of 0.25, we needed a NSCLC patient sample size of 630. We set nine datasets performed by platform GPL570 (including 54,675 probes) as training cohort (n = 843). Since GSE30219 (Rousseaux et al., 2013) was the largest single study including all cancer stages and all cancer cell types, we used it as a testing cohort in combination with GSE8894 (Lee et al., 2008), which only contained recurrence-free survival (RFS) data. Six other datasets collected on different platforms were also used for verification (Takeuchi et al., 2006, Raponi et al., 2006, Director's Challenge Consortium for the Molecular Classification of Lung, A., et al., 2008, Zhu et al., 2010, Sato et al., 2013, Tang et al., 2013). We downloaded all available original CEL files and normalized them with Robust Multichip Average from Affymetrix Expression Console.

### 2.2 Combining Nine Datasets in Training Cohort and Three Datasets in Testing Cohort

**Fig. 1:**
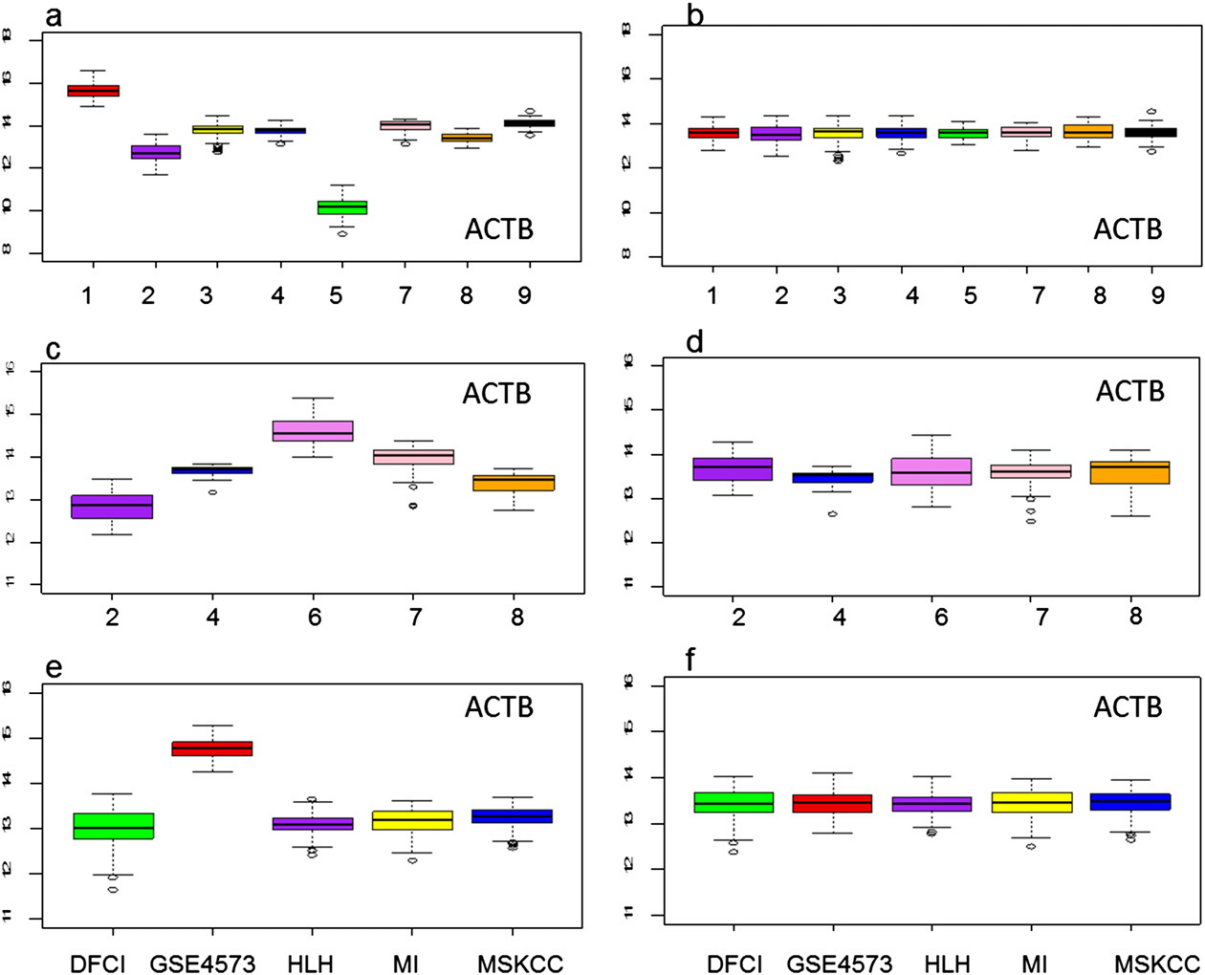
Comparison of batch effects among multiple datasets of NSCLC before and after COMBAT. a. The expression levels of ACTB showed large batch effects among eight datasets (1: GSE3141, n = 111; 2: GSE19188, n = 91; 3: GSE37745, n = 196; 4: GSE31210, n = 226; 5: GSE29013, n = 55; 7: GSE19804, n = 60; 8: GSE18842, n = 46; 9: GSE10245, n = 58) of NSCLC in training cohort before COMBAT. b. The batch effects among eight datasets of NSCLC in training cohort have been completely removed by COMBAT. c. There were large batch effects among five healthy lung control or tumor surrounding normal tissue datasets (2: GSE19188, n = 65; 4: GSE31210, n = 20; 6: GSE1643, n = 40; 7: GSE19804, n = 60; 8: GSE18842, n = 45). d. The batch effects among five healthy lung control or tumor surrounding normal tissue datasets in training cohort have been eliminated by COMBAT. e. There were some batch effects among five datasets (DFCI, HLM, MI, MSKCC and GSE4573, n = 659) of NSCLC in testing cohort before COMBAT. f. The batch effects among five datasets of NSCLC in testing cohort were completely eliminated by COMBAT. Bottom, middle, and top lines of each box corresponded to the 25th percentile, the 50th percentile (median), and the 75th percentile, respectively. The caps showed minimum and maximum values excluding outliers.

The optimal way of grouping the patient data was to combine all 2738 available samples together and randomize them into two groups: the training cohort and the testing cohort. However, due to the fact that the available datasets were performed on different platforms and contained batch effects, we were compelled to adopt another approach. Although the platform was the same for some datasets, it was impossible to combine them directly due to large batch effects among different datasets (Fig. 1a, c, e). To remove these batch effects, we decided to use COMBAT because it outperformed other available methods (Chen et al., 2011). Using the COMBAT methodology described previously in Chen, C. et al., we standardized the nine datasets we combined for the training cohort (Chen et al., 2011). Similarly we combined three GPL96 (22,283 probes) datasets for the largest testing cohort. GSE42127 (Tang et al., 2013) and GSE41271 (Sato et al., 2013) were obtained with platform GPL6884 (48,803 probes), and to avoid loss of any gene information, we did not perform data merging among different platforms.

### 2.3 Significance Analysis of Differentially Expressed Genes

Siggenes was used to identify the differentially expressed genes as previously described (Chen et al., 2012). Since multiple two-group comparisons may introduce some errors, we further compared the three groups simultaneously, and then found the gene expression differences that were common to all comparisons (Fig. 3).

### 2.4 Univariate & Multivariate Analyses (Accelerated Failure Time Model, AFT)

While some studies published overall survival (OS) data that exceeded 5 years of follow-up (Botling et al., 2013, Arriagada et al., 2010), others truncated the data at 5 years (Raponi et al., 2006, Director's Challenge Consortium for the Molecular Classification of Lung, A., et al., 2008, Lu et al., 2010, Okayama et al., 2012, Rousseaux et al., 2013). To generate a more reliable model, we analyzed all available data. The drawback of OS data is that as time passes it can be influenced by many other factors than the cancer itself. To account for the effect of time on OS, we used the AFT model for univariate & multivariate analyses.

### 2.5 Kaplan–Meier Analysis

Kaplan–Meier curve takes into account right-censoring, and all of the NSCLC datasets were right-censored data. We performed Kaplan–Meier analyses and chi-square (*χ*^2^) tests were used to determine significant differences in R.

### 2.6 Converting Data from Two Channels to Single Channel

There was only one dataset (GSE11969 (Takeuchi et al., 2006)) in testing cohort which was performed with Agilent's two-channel array GPL7015. Two-channel array introduced a reference RNA (labeled with Cyanine-3: Cy3) to compare the samples (labeled with Cyanine-5: Cy5) and exported the ratios of Cy5/Cy3 as follows:

(1) $$E_{\mathrm{two}}=\log_{10}\left(\frac{Cy5}{Cy3}\right)\;=\log_{10}\left(\frac{{\mathrm{GeneX}}_{\mathrm{NSCLC}}\;}{{\mathrm{GeneX}}_{\mathrm{reference}}}\right)\;=\log_{10}\left({\mathrm{GeneX}}_{\mathrm{NSCLC}}\right)-\log_{10}\left({\mathrm{GeneX}}_{\mathrm{reference}}\right)\text{.}$$

All single channel data are transformed into log_2_ values:

(2) $$E_{\mathrm{single}}=\log_{2}\left({\mathrm{GeneX}}_{\mathrm{NSCLC}}\right)=\log_{10}\left({\mathrm{GeneX}}_{\mathrm{NSCLC}}\right)/\log_{10}2\text{.}$$

Combine functions (1), (2):

(3) $$E_{\mathrm{single}}=\;\left(E_{\mathrm{two}}+\;\log_{10}\left({\mathrm{GeneX}}_{\mathrm{reference}}\right)\right)\;/\;\log_{10}2$$

where E_two_ was normalized log_10_ ratio of Cy5/Cy3 representing sample/reference. E_single_ was normalized log_2_ values of intensity only representing sample. GeneX_NSCLC_ was intensity value of sample. GeneX_reference_ was intensity value of reference RNA.

In GSE11969, total RNA from 20 lung cell lines representing all major histological types of NSCLC was reference. We were able to use the mean expression value of any gene from one-channel of NSCLC cell lines to estimate the log_10_(GeneX_reference_). Using function (3), it was easy to transform all log_10_ ratios of two-channel data into one-channel data.

## 3 Results

### 3.1 Removal of Large Batch Effects

The housekeeping gene Beta-actin (ACTB) expression showed that there were large batch effects due to institutional variations among the training datasets (Fig. 1a, c). The biggest variation was observed between the datasets of study 1 (GSE3141 (Bild et al., 2006)) and study 5 (GSE29013 (Xie et al., 2011)), which showed more than a 32 fold-difference in expression levels. We observed similar batch effects in our testing cohort (Fig. 1e). After application of COMBAT, the batch effects were eliminated (Fig. 1b, d, f).

### 3.2 Analysis of NSCLC Survival Distributions Suggests Multiple Genes Govern Survival

**Fig. 2:**
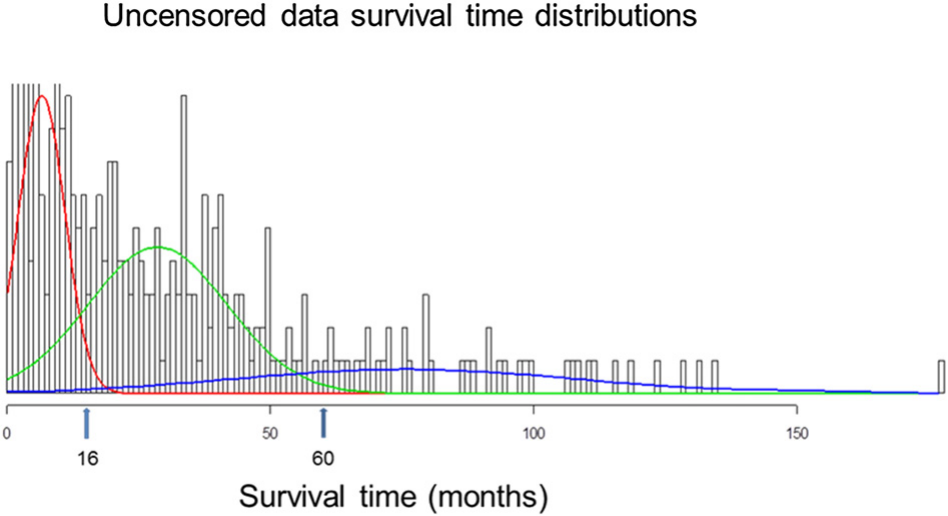
The distributions of overall survival time (OST, months) of NSCLC. The histograms showed the frequencies of OST for 306 of deaths in training cohort. The color curves are the fits with three normal distributions. The arrows show the best cutting off values (16 m and 60 m) for three survival groups.

The overall survival (OS) of the 306 NSCLC patients that died before the studies concluded exhibited a three-peak distribution. We were able to fit data to three normal distributions and sort patients into three different groups: good outcome (> 60 months), intermediate outcome (16–60 months), and poor outcome (< 16 months; Fig. 2). The distributions suggested that OS was influenced by multiple genes, and consequently, we predicted that there might be at least six or more genes that could be used to model OS.

### 3.3 Differential Gene Expression Analysis Yields Seven-gene Score

**Fig. 3:**
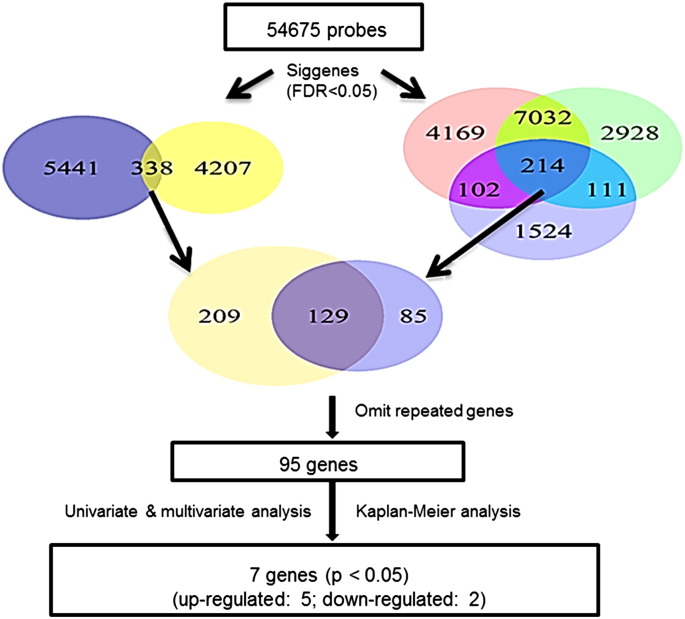
Strategies for gene screening. We have performed Siggenes analysis for multiple two-group comparisons (H vs Ca; N vs Ca and poor (OST < 16 m) vs good clinical outcome (OST > 60 m)) and two three-group comparisons (H-vs-N-vs-Ca and poor (OST < 16 m) vs good clinical outcome (OST > 60 m) vs intermediate subgroup (16 m < OST < 60 m)). The FDR are less than 0.01 or < 0.05. From a total of 54,675 probes, we have identified 11,571 probes differentially expressed between the two groups (H vs Ca), 10,285 probes differentially expressed between N and Ca samples and 1951 probes differentially expressed between poor clinical outcome group and good clinical outcome group. Intersecting the three sets of differentially expressed probes, we have identified 214 common probes (Fig. 3 right). Among H, N and Ca three groups, we have identified 5779 probes and 4545 differentially expressed probes among different clinical outcome groups. Intersecting the two sets of differentially expressed probes, we have also identified 338 common probes (Fig. 3 left). Intersecting the two sets of differentially expressed probes from two different strategies, we have identified 129 common probes. There are only 95 of common genes differentially expressed excluding 34 probes that shared the same gene names among the 129 common probes. We have performed univariate analysis (AFT model) for all of those 95 genes. For the genes with p value less than 0.01 we have further performed multivariate analysis and Kaplan–Meier analyses. Using 0.05 as p cutting off values, we have finally identified seven genes (included 5 up-regulated and 2 down-regulated genes).

**Fig. 4:**
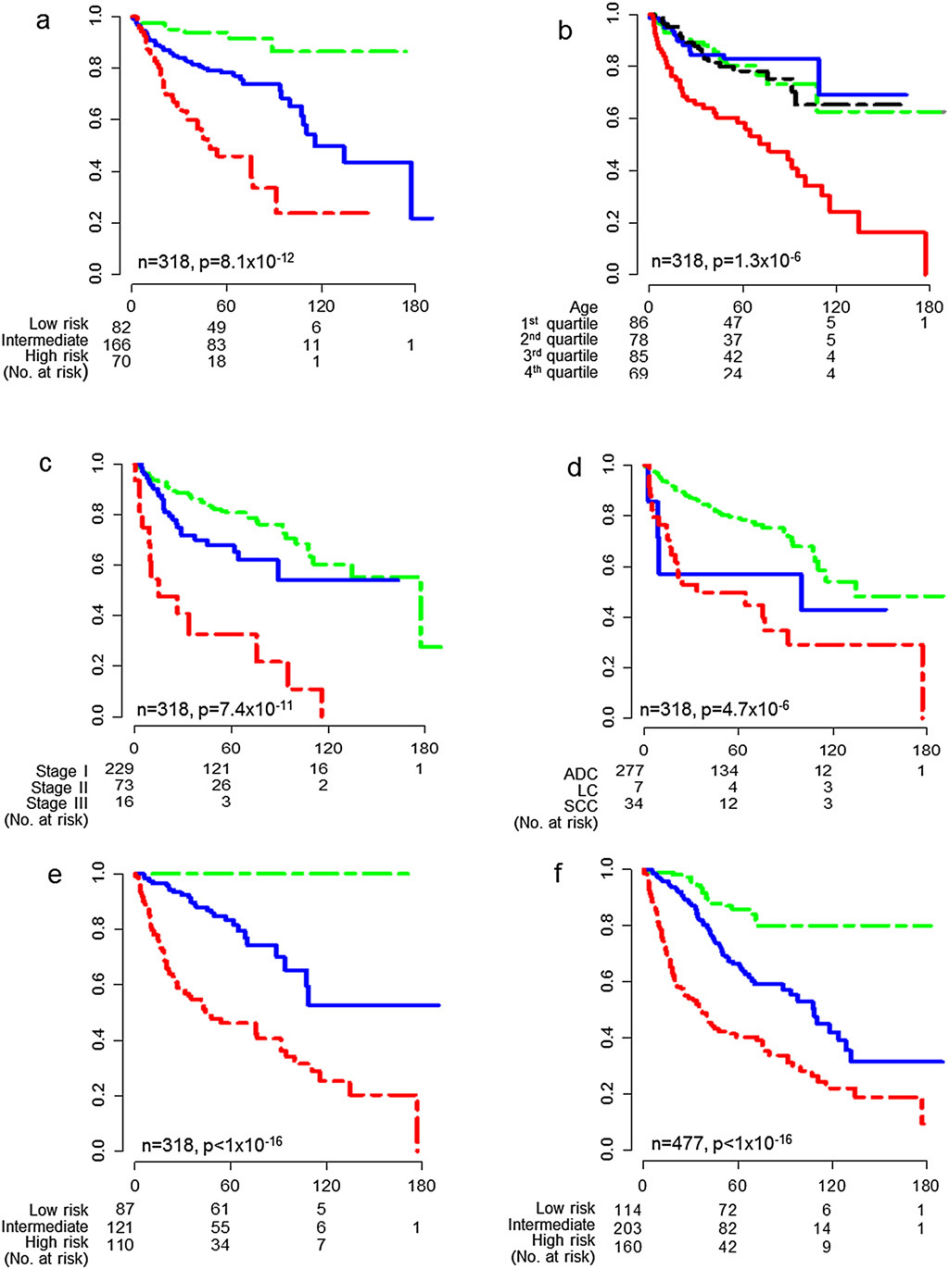
Kaplan–Meier analysis of OS on training cohort. a. Using seven-gene score to predict OS in three stages and three cell types without ACT. b. Using age to predict OS in three stages and three cell types without ACT. The green, blue, black and red lines correspond to the first, second, third and fourth quartiles respectively. c. Using stages to predict OS in three cell types without ACT. The green, blue and red lines correspond to the stages I, II and III separately. d. Using cell types to predict OS in three stages without ACT. The green, blue and red lines correspond to ADC, LC and SCC respectively. e. LCPI defines low, intermediate and high risk subgroups in training cohort without ACT for OS. f. LCPI defines low, intermediate and high risk subgroups in training cohort with ACT for OS. In a, e, and f, green, blue and red lines correspond to low, intermediate and high risk subgroups respectively. The x-axis is the survival time (months), the y-axis is survival probability.

To generate a multi-gene model for OS, we sought relevant genes using the Siggenes in R, and compared the samples in our training cohort (n = 1073; Fig. 3). Most of the studies from which we obtained our datasets used the tissues surrounding the lung tumors from the NSCLC patients (N) as a control as opposed to the more difficult to obtain normal lung tissues from the healthy lung (H). When we compared H and N, we found that there were 2555 of genes differentially expressed between H and N. This indicated that the tissue surrounding lung tumors was very different molecularly from actual healthy tissue. For comparison to cancerous lung tissue (Ca), the best control should be H and not N. However, we were restricted by the available data as many samples (170) in our datasets were surrounding tissue (N), and only 60 samples were healthy tissue samples (H). Thus, we employed an alternative approach and we used both H and N as separate controls. If a biomarker for NSCLC survival is reliable, it should be consistently different in the comparisons H vs Ca and N vs Ca. Since multiple two-group comparisons may introduce errors, we further compared the three groups simultaneously, and then found the gene expression differences that were common to all comparisons. This comparison revealed the genes that were differentially expressed for lung cancer tumors, but this did not necessarily mean that they were all related to survival. We then analyzed the different survival groups using a similar comparison, and overlaid the probes of interest from the first comparison (214 probes) with those from the second comparison (338 probes), and found 129 common probes that were differentially expressed among all groups. We conducted univariate, multivariate, and Kaplan–Meier analyses and found 7 significant genes (Fig. 3, Table 2. The p values in univariate, multivariate, and Kaplan–Meier analyses were less than 0.05.). We generated a seven-gene score for each patient by adding the values of each coefficient (from multivariate coxph model) multiplied by its respective gene expression value (seven-gene score = b_1_gene_1_ + b_2_gene_2_ + … + b_7_gene_7_). In our training cohort, survival data with all clinical parameters was only available for 477 patient samples. To avoid any confounding effect of ACT, we excluded any patient that received ACT or an unknown treatment (n = 159). Applying this score in Kaplan–Meier analysis, we separated patients (n = 318) into distinct three groups by best cutoffs (Fig. 4a).

### 3.4 Seven-gene Score, Age and Stage are Independent Predictors

Multivariate analysis of available clinical parameters (age, gender, stage and cell type) suggested that cancer age, stage, and cell type might be independent predictors of survival (Table 3). However, Kaplan–Meier analyses using these factors were only able to separate the patient samples into two distinct groups (Fig. 4b–d). When we introduced the seven-gene score into our multivariate analysis of clinical parameters, we found that while age and stage remained independent, cell type was no longer significant. Furthermore, the hazard ratio (HR) and p-value indicate that the seven-gene score is the most powerful independent predictor (Table 3).

### 3.5 Seven-gene Score, Age and Stage Constitute LCPI

Having determined the seven-gene score, age and stage as independent predictors of OS, we were able to generate survival functions:

(4) $$S\left(t\right)=e^{-\lambda t}$$

(5) $$\mathrm{LCPI}=\lambda =b_{1}{\mathrm{gene}}_{1}+b_{2}{\mathrm{gene}}_{2}+\ldots +b_{7}{\mathrm{gene}}_{7}+b_{8}\mathrm{age}+b_{9}\mathrm{stage}$$

where S(t) is the survival probability before time t; λ is HR; LCPI is the lung cancer prediction index; b_1_ to b_9_ are coefficients calculated from the data in our training cohort with coxph model, they are 0.45(VANGL1), 0.36(GNAI3), 0.30(CTSB), − 0.44(ANKRD11), − 0.49(ITPKB), 0.03(KIAA0101), 0.05(PLOD2), 0.03(age) and 0.69(stage) separately, and remain constant in all LCPI calculations; gene_1_ to gene_7_ are the log_2_ values of GEP; age is the real age(# in years); and stage values are 0 to 3 (stage IA = 0, stages IB–IIB = 1, stages IIIA–IV = 3). To output the LCPI, we input the expression values of the seven genes (gene_1_, gene_2_, gene_3_, etc. log_2_ values), as well as the age (# in years), and stage of the cancer (0 to 3). Using above function (5), we were able to calculate the LCPI score for any patient and predict his/her OS (function (4)). Lower LCPI corresponded with higher survival probability while higher scores correspond to lower probability of survival, and higher likelihood of death and cancer recurrence. The cutoff value was the same as that in training cohort for the data from the same platform. For the data from different platforms, we adjusted it to the best cutoff.

We separated our training cohort (n = 318) into three clearly distinct groups using LCPI (Fig. 4e). At ten years after surgery, the survival probability of the low risk group was 100%, and remained the same even after 15 years. In the intermediate risk group, the survival probability at 15 years was 53 ± 10% (p < 0.001). The survival probability of the high-risk group was less than 20% at 15 years. From the analysis of the training cohort, we are able to obtain the best cutoff values for each risk group, and then apply them to the testing cohorts as pre-specified cutoffs. For datasets obtained using different platforms, the best cutoff calculation was performed to obtain cutoff values for each risk group.

### 3.6 ACT Negatively Impacts OS for Low and Intermediate Risk Groups

To discern whether ACT influences OS, we included data from patients that received ACT or an unknown treatment and applied the LCPI (n = 477). The fact that we observed similar separation of risk groups with or without patients treated with ACT or unknown confirmed that the exclusion does not affect the LCPI model's ability to assign patients to risk groups (Fig. 4f). At 15 years after surgery, we observed lower survival probabilities for both the low and intermediate risk groups, which were 80 ± 5% and 30 ± 10% (p < 0.05), respectively. Comparing to the cohort that did not receive treatment after surgery, the cohort that included patients who received ACT or an unknown treatment showed significant decreases in survival probabilities for the low and intermediate risk groups (80 ± 5% vs. 100%, p < 0.001; 30 ± 10% vs. 53 ± 10%, p < 0.05). This suggests the possibility that ACT may have a negative impact on individuals with low or intermediate risk, as determined by the LCPI.

**Fig. 5:**
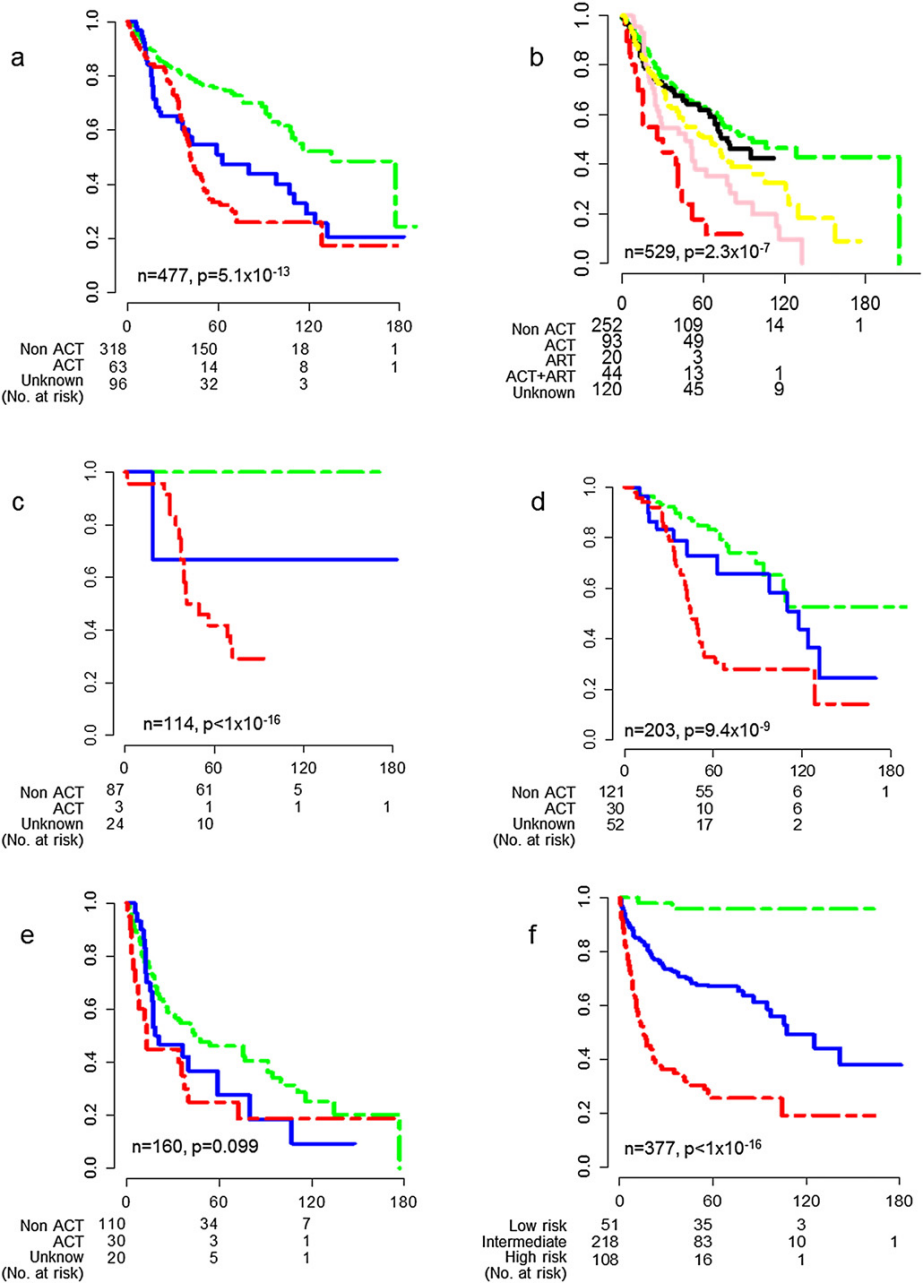
Effects of ACT or ART on NSCLC in training and testing cohorts and LCPI for RFS. a. The OS probabilities in both ACT (red) and unknown (blue) subgroups were markedly decreased comparing to non-ACT subgroup (green) in training cohort. b. The OS probability in ART (red) subgroup was the lowest comparing to other subgroups in testing cohort. On contrary, the OS probability in non-adjuvant treatment (green) subgroup was the highest. The OS probabilities in ACT (black), ACT + ART (pink) and unknown (yellow) subgroups were lower than non-adjuvant treatment subgroup (green), but higher than ART subgroup (red). c. In low risk subgroup defined by LCPI in training cohort, all the patients in non-ACT subgroup (green) had high up to 100% of survival probabilities at 15 years, but the survival probabilities in ACT (blue) or unknown subgroups were sharply dropped. d. In intermediate risk subgroup defined by LCPI in training cohort, ACT (blue) had no benefit even made it worse at longer follow-up time compared to non-ACT (green). The survival probability in unknown subgroup (red) was severely dropped at any time points. e. In high risk subgroup defined by LCPI in training cohort, the survival probabilities in ACT (blue) and unknown (red) subgroups were similar to non-ACT subgroup (green). The x-axis is the survival time (months), the y-axis is survival probability. f. LCPI defined low, intermediate and high risk subgroups in training cohort for RFS.

To further explore the impact of ACT on OS, we separated the patient pool (n = 477) into non-ACT, ACT and unknown treatment groups. The non-ACT group exhibited the best OS, while the ACT group or surgery plus unknown treatment showed worse OS (Fig. 5a; p < 0.001). We verified this outcome with the testing cohort (n = 529) and observed similar results (Fig. 5b, p < 0.001).

Given the effect we observed in the training and testing cohorts, we were curious whether ACT equally affected each LCPI risk group, so we analyzed the survival of each risk group in our training cohort separately. While ACT did not influence the survival of the patients in the high risk group, it was detrimental for patients in the low and intermediate risk groups (Fig. 5c–e).

Since OS may sometimes be influenced by other factors, we analyzed the RFS data as well. Recurrence after surgical resection is the main reason for the early death of NSCLC patients, and RFS is more reliable than OS. Recurrence data was only available for 377 of the 477 patients in our training cohort, and after application of LCPI, we were again able to distinguish the three risk groups (Fig. 5f; p < 0.001). The recurrence data supports our analysis of the OS data.

### 3.7 Verification of LCPI in the Largest Multiple Institutions Dataset from the USA and Canada

**Fig. 6:**
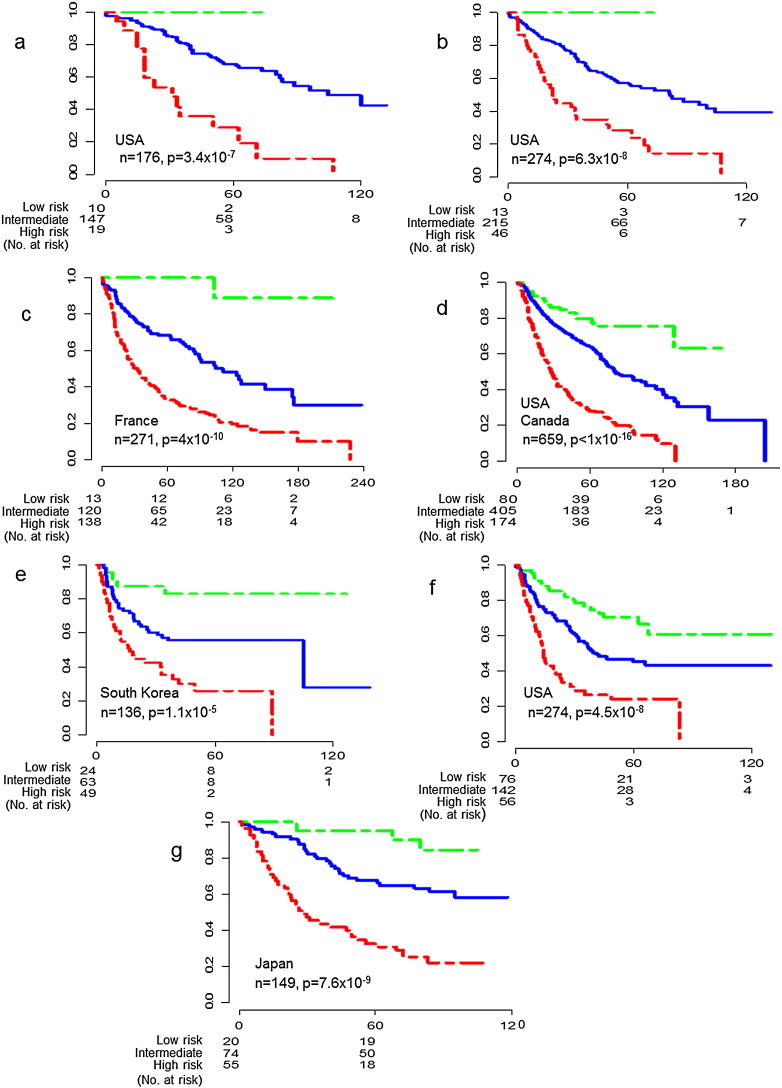
Verification of LCPI in multiple large NSCLC datasets including all stages and all cell types from multiple countries. a. OS, dataset GSE42127, n = 176, including two cell types, all stages and 49 ACT, from the USA. b. OS, dataset GSE41271, n = 274, including seven cell types, all stages and 49 ACT, from the USA. c. OS, dataset GSE30219, n = 271, including seven cell types, all stages from France. d. OS, Integrated datasets (DFCI, HLM, MI, MSKCC and GSE4573), n = 659, including three cell types, three stages (I–III), 137 ACT and 64 ART, from the USA & Canada. e. RFS, dataset GSE8894, n = 136, including two cell types and all stages, from South Korea. f. RFS, dataset GSE41271, n = 274, including seven cell types, all stages and 49 ACT, from the USA. g. OS, two-channel dataset GSE11969, n = 149, including five cell types and three stages (I–III), from Japan. In a, b, c, d, e, f, and g, green, blue and red lines correspond to low, intermediate and high risk subgroups defined by LCPI respectively. The x-axis is the survival time (months), the y-axis is survival probability.

After integrating Jacob-00182 (Director's Challenge Consortium for the Molecular Classification of Lung, A. et al., 2008), GSE14814 (Zhu et al., 2010) and GSE4573 (Raponi et al., 2006) datasets with COMBAT, we produced the second largest multiple institution dataset for NSCLC, which included all stages, three cell types and post-surgery ACT or ART from seven institutions in the United States and Canada without batch effects (n = 659). This dataset was obtained using the Affymetrix platform GPL96, which differed from our training cohort, so we verified the power of LCPI by adjusting it to the best cutoff. Fig. 6d showed that using besting cutoff values for this cohort performed using this platform, LCPI was able to separate the 659 NSCLC patients into three distinct risk subgroups. The OS probabilities in high risk subgroup at five years and 10 years were 28% and 9.5% respectively. All patients died before 130 months. The OS probabilities in intermediate risk subgroup at five years, 10 years and 15 years were 64%, 39% and 23%. The above results were very similar to the results in 477 of training dataset included ACT and unknown patients. But the OS probabilities in low risk subgroup at 5 years, 10 years and 15 years were 80%, 76% and 63% which were lower than that in 477 of training dataset. Given our previous analysis (Fig. 4, Fig. 5), it is possible that these differences may be attributable to patients with ART and/or ACT (Fig. 5b). However, further study would be required to confirm the effect of post-surgical ACT for NSCLC. The above results indicated that LCPI was able to work in multiple institution dataset of NSCLC including all stages, three cell types and different adjuvant treatments (ACT and/or ART).

### 3.8 Verification of LCPI in USA Dataset GSE42127

The samples in dataset GSE42127 (Tang et al., 2013) were from MD Anderson Cancer Center in Texas, United States. In this independent testing cohort, 133 patients were adenocarcinomas (ADC) and 43 patients were afflicted with squamous cell carcinomas (SCC). Forty-nine patients received ACT (mainly Carboplatin plus Taxanes) and 127 patients did not receive ACT. The patient sample included patients with cancer stages I, II, III and IV. We applied LCPI to this dataset, and since this cohort differed in platform, we used the best cutoff values to separate patients into different risk groups. Fig. 6a showed that LCPI was able to separate this cohort into three distinct subgroups (low, intermediate and high risk subgroups) similar to that in training cohort. The OS probability of low risk subgroup was up to 100% at 80 months, and the OS probability of intermediate risk subgroup was great than 40% at 10 years while all of the patients in high risk subgroup died before 10 years.

### 3.9 Verification of LCPI in the Largest Single Institution Dataset GSE41271 from the USA

To date GSE41271 (Sato et al., 2013), which included 176 samples from GSE42127 (Tang et al., 2013), was the largest NSCLC dataset from single institution in the United States (n = 275). The patients in this testing cohort belong to four different races (Caucasian, African American, Hispanic and Asian), and the clinical stages in this cohort were from IA to IV. There were 184 ADC patients, 80 SCC patients, and 10 patients that had five over rare cell types. One patient sample did not have the data necessary for analysis, and was not included. Using LCPI we performed Kaplan–Meier analyses for this testing cohort, which was performed with a different platform, by adjusting to the best cutoff. Fig. 6b showed that the results were very similar to that of the testing cohort GSE42127. The OS probability of low risk subgroup was up to 100% at 80 months, and the OS probability of intermediate risk subgroup was about 40% at 10 years while all of the patients in high risk subgroup died before 10 years. That suggested even in large dataset that included different races, some use of ACT, all stages and all cell types of NSCLC, LCPI still worked very well for identifying three different risk subgroups.

### 3.10 Verification of LCPI in the Largest Single Institution Dataset GSE30219 from France

GSE30219 (Rousseaux et al., 2013) was the largest single institution dataset from France even excluding the control (n = 14) and small cell lung cancer samples (n = 22), which were not relevant to our study. There were 271 of NSCLC including all stages and seven cell types in this testing cohort. The data were obtained using the same platform as the training data, so we were able to apply LCPI to this cohort with pre-specified cutoff or the same cutoff value as that of the training cohort (6.83, 8.19). Fig. 6c showed that LCPI was able to separate this cohort into three distinct subgroups (low, intermediate and high risk subgroups) similar to that in training cohort and testing cohorts (GSE42127 (Tang et al., 2013), GSE41271 (Sato et al., 2013)). The OS probability of low risk subgroup was up to 100% at six years, stable at 89% from 10 years to over 18 years. The OS probability of intermediate risk subgroup was greater than 40% at 10 years and greater than 30% at 18 years. While the OS probabilities in high risk subgroup at any given time point were significantly lower than the other two subgroups. This was a single dataset, and since we did not need to combine it with another, we did not perform COMBAT. Even without the use of COMBAT, LCPI still worked very well for identifying three different risk subgroups for the France dataset, which included all stages and all cell types of NSCLC.

### 3.11 Verification of LCPI to Predict RFS in South Korea Dataset GSE8894

Recurrences after surgical resection are the main reasons for the early deaths of NSCLC patients. RFS tends to be more reliable than OS because it is not affected by nonspecific deaths. If our LCPI model is reliable, it should work for both OS and RFS in multiple countries. This RFS dataset GSE8894 (Lee et al., 2008) from South Korea included 138 of NSCLC patients (two cell types). Two patients were missing the necessary data, and were thus excluded. The platform was the same as training cohort, but the stage information was not available. Then we applied LCPI without inputting data about cancer stage in 136 of NSCLC patients and defined risk groups by best cutoff. Although we did not have cancer stage information, our model was still able to define risk groups for the RFS data (Fig. 6e). The 136 of patients were separated into three different risk subgroups. All patients in high risk subgroup were recurrent before eight years while the probability of RFS in intermediate risk and low risk subgroups were great than 55% and 83% respectively at eight years.

### 3.12 Verification of LCPI to Predict RFS in the Largest Single Institution Dataset GSE41271 from the USA

The largest NSCLC dataset for OS and RFS from a single institution in the United States (n = 275) was GSE41271 (Sato et al., 2013). One patient sample did not possess the complete data required for analysis, and was excluded from our study. We applied LCPI to the 274 NSCLC patients in this cohort, which included RFS data from patients with all stage and all cell types. The cutoff value was the same as that for the OS analysis (Fig. 6b). LCPI separated the dataset into three significantly different risk subgroups (Fig. 6f). All patients in high risk subgroup experienced cancer recurrence before eight years while the probability of RFS in intermediate risk and low risk subgroups were great than 52% and 100% separately at five years. These results provide further support for the LCPI model's ability to separate low, intermediate and high risk subgroups for overall survival as well as recurrence datasets.

### 3.13 Verification of LCPI to Predict OS in Two-channel Dataset GSE11969 from Japan

So far we have verified LCPI in all available NSCLC single channel array datasets from multiple countries. Some of datasets were performed with Agilent's two-channel array GPL7015 platform instead of single-channel array. There were 149 NSCLC patients in the Japanese cohort, GSE11969 (Takeuchi et al., 2006), which included IA to IIIB and five cell types. Using function (3) we were able to transform two-channel array data into single channel data and get the LCPI score. Here we also defined risk group cutoffs to best cutoff. We showed that LCPI was able to separate this cohort into three different risk subgroups (Fig. 6g). The OS probabilities in the low, intermediate and high risk subgroups were 95%, 68% and 32% at 5 years and 84%, 58% and 22% at about 10 years respectively.

In summary, the most important aspect of any predictive model is its validation. To confirm the power of LCPI, we verified its ability to predict survival time using multiple datasets of NSCLC (n = 1665, all stages and multiple cell types) from five countries (Fig. 6).

GSE42127 (n = 176) and GSE41271 (n = 274) included patients with all four stages and multiple cell types, some of which received ACT after operation. Application of LCPI to the OS data allowed us to separate these cohorts into the same risk groups we observed in the training cohort (Fig. 6a, b). We also analyzed the available RFS data (n = 274) using LCPI. The recurrence analysis of the testing cohort further verified the predictive power of LCPI (Fig. 6f).

To assess whether LCPI can be accurately applied to data collected from different countries, we applied it to datasets GSE30219 (n = 271, France), GSE8894 (n = 136, South Korea), GSE11969 (n = 149, Japan), and the combined datasets Jacob-00182, GSE14814 and GSE4573 (n = 659, the USA and Canada). After application of LCPI to the OS data of each dataset, we were able to observe distinct risk groups for all available testing cohorts (Fig. 6c, d, g). Similarly, we were able to predict the RFS for GSE8894 and separate patients into different risk groups (Fig. 6e). The fact that LCPI consistently predicted high, intermediate, and low risk groups for all the tested datasets demonstrates its reliability.

## 4 Discussion

We have proposed a multigene model (LCPI), which incorporates seven differentially expressed genes, age and stage, to predict clinical outcome. Utilizing the LCPI, we were able to separate patients into three distinct groups with different survival probabilities (Fig. 4, Fig. 6). Aided by this model, clinicians will be able to personalize post-surgical treatment for NSCLC patients. Low risk individuals have very high survival probabilities and may not require any further treatment beyond regular observation (Fig. 4e). The average age for patients that received surgery for NSCLC was around 62, and our model showed that the low risk individuals could survive more than 15 years after surgery. If we consider that the average world life expectancy is around 70–80 years old, then the average patient in the low risk group could expect to live out his/her full life expectancy after surgery. In fact, our data suggests that for patients in the low or intermediate risk groups, post-surgery treatment like ACT may actually decrease survival probabilities (Fig. 4e, f). For patients that have high risk, as determined by LCPI, surgery is insufficient. Based on the patient's survival probability, clinicians can determine whether to use conservative, aggressive, or experimental treatment strategies following surgical resection.

Efforts to find a predictive model for lung cancer have been underway since 2001 (Bhattacharjee et al., 2001) and at present, more than 17 independent NSCLC gene expression datasets and their respective predictive models have been published. However, while these models span the spectrum between a single gene to hundreds of genes, their predictive abilities are limited by small sample sizes and institutional variations. In order to account for sample size and increase the power of our model, we combined nine different datasets with NSCLC samples and control samples for our training cohort. To account for institutional variation, we used COMBAT to completely eliminate the batch effects observed among the different datasets (Fig. 1). Using this strategy, we generated two of our largest datasets, a training cohort of n = 1073 and a testing cohort of n = 659. From the training cohort, we created a LCPI capable of predicting individual survival probabilities using the expression levels of seven genes, age, and stage. Since the success of a predictive model is determined by its verification, we tested our model using several independent datasets collected from multiple countries (Fig. 6). These testing cohorts contained samples from patients with multiple stages and cell types. The fact that our model was able to separate these patients into three distinct risk groups regardless of cancer stage, cell type, and country of origin, illustrates the exceptional reliability and predictive capacity of the LCPI.

Shedden et al. provided one of the largest gene-expression datasets for NSCLC in 2008 (Director's Challenge Consortium for the Molecular Classification of Lung, A. et al., 2008). After the analysis of several different methodologies for the prediction of tumor biology and the inference of patient survival, they concluded that the subject outcome was best predicted using 100 gene clusters with clinical parameters. In 2012, Okayama et al. proposed a similarly large predictive model using 174-gene signatures (Okayama et al., 2012). Regardless of predictive accuracy, however, the collection and analysis of hundreds of genes to infer patient prognosis are economically unfeasible and difficult to apply in practice. Furthermore, compared to many of published models for NSCLC, which have been developed from data truncated at 60 months, we've shown in our model verification that our seven-gene model is capable of clearly distinguishing patient survival groups from uncensored data collected over 200 months (Fig. 6c).

The postoperative use of ACT is the standard of care for the management of some stages of NSCLC. The benefits of ACT, however, remain debatable. Some studies have shown that NSCLC patients treated with ACT have prolonged survival (Winton et al., 2005, Douillard et al., 2006, Soria et al., 2013), while some of them failed to observe any overall survival benefit with ACT (Waller et al., 2004, Scagliotti and Novello, 2003). Five of the largest adjuvant trials to date include the following: (1) National Cancer Institute of Canada (NCIC) JBR.10 (n = 482), (2) Adjuvant Navelbine International Trialist Association (ANITA, n = 840), (3) Big lung trial (BLT), (4) International Trialist Association Trial (IALT, n = 1867), and (5) Adjuvant Lung Project Italy (ALPI) (Patel and Wakelee, 2011b). The NCIC JBR.10 (Winton et al., 2005) and the ANITA trials (Douillard et al., 2006) demonstrated OS benefit and the survival advantage did not diminish over time at seven year follow-up. The IALT showed a slightly improvement in the five-year survival rate of 4% with adjuvant chemotherapy (Arriagada et al., 2004). The BLT (Waller et al., 2004) and the ALPI (Scagliotti and Novello, 2003) trials were negative. Another dataset of 2194 patients (1313 bevacizumab; 881 controls) from four phase II and III trials showed that bevacizumab significantly prolonged OS and RFS (Soria et al., 2013). The NSCLC Meta-analysis Collaborative Group published a paper in Lancet in April, 2010, which summarized 34 trials, showed the benefit of adjuvant therapy was undeniable at 5 years, the improvement was slight (4%) at 5 years (NSCLC Meta-analysis Collaborative Group, 2010). Contributing to the ongoing dialog regarding the effectiveness of ACT, our analysis suggests that post-operative ACT treatment may have a detrimental effect on individuals that have low or intermediate risk, as determined by LCPI (Fig. 4e, f). While further investigation is necessary to confirm our observation, it highlights a pressing need to determine the effectiveness of ACT as a treatment for low-risk NSCLC. In some cases, postoperative treatment is unnecessary, and an accurate predictive model can help clinicians individualize treatments for NSCLC.

We conclude that survival time of NSCLC is a quantitative trait. The seven genes, age and stages together determine the survival probability at 10 and 15 years. LCPI is able to simultaneously define three risk subgroups for all stages and multiple cell types of NSCLC. Based on our analysis of patients defined to be low risk by LCPI, surgical resection may be sufficient to maximize overall survival and recurrence free survival.

## Funding

No funding.

## Author Contributions

Conception and design: Tiehua Chen, Luming Chen.

Collection and assembly of data: Tiehua Chen, Luming Chen.

Data analysis and interpretation: Tiehua Chen, Luming Chen.

Manuscript writing: Luming Chen, Tiehua Chen.

Final approval of manuscript: Tiehua Chen, Luming Chen.

## Figures and Tables

**Table 1:** Summary of 17 GEP Datasets of NSCLC.

Ref no.	GSE ID	First author	Number of genes used in author's model	Stages	Cell types	Training/test	Survival probability of low risk group at 5 years	Survival probability of low risk group at 15 years	Data truncated at 5 years
5	3141	Bild AH	NA	NA	ADC, SCC	Training	68%±	NA	NA
6	11969	Takeuchi T		I–III	ADC	TE	78%±	NA	No
7	1643	Gruber MP	Healthy	NA	NA	Training	NA	NA	NA
8	4573	Raponi M	100	I	SCC, ADC	Test	NA	NA	Yes
9	NA	Shedden K	100	I–III	ADC	Test	62%±	NA	Yes
10	8894	Lee ES	6	I–III	ADC, SCC	Test	60%±	NA	No
11	10245	Kuner R	17	I–III	ADC; SCC	Training	NA	NA	No
12	19804	Lu TP	1	I–IV	ADC	Training	22%±; 45% ± _60%±	NA	Yes
13	14814	Zhu CQ	15	I–II	ADC, SCC	Test	90%±	NA	No (9 years)
14	19188	Hou J	17	I–IV	ALL	Training	58% ± _68%±	NA	No (> 10 years)
15	18842	Sanchez-Palencia A	92	I–IV	ADC, SCC	Training	NA	NA	No
16	29013	Xie Y	59	I	ADC, SCC	Training	46% ± _51%±	NA	Yes (7 years)
17	31210	Okayama H	9	I–II	ADC	Training	84%±	NA	Yes (98–2008)
18	37745	Botling J	14(1)	NA	ADC, SCC, LCC	Training	61%±	20%±	No (95–2005)
19	30219	Rousseaux S	26	I–IV	ALL	Test	66%±	NA	Yes (Max: 240 M)
20	41271	Sato M	171	I–III	ADC, SCC	Test	70%±	NA	No
21	42127	Tang H	18(12)	I–III	ADC	Test	78%±	NA	No (96–2007)

**Table 2:** The name, ID, location and aliases of seven common genes.

Name	Gene ID	Location	Aliases
ANKRD11	29123	Chromosome 16, NC_000016.10 (89267619..89490561, complement)	ANCO-1, ANCO1, LZ16, T13
CTSB	1508	Chromosome 8, NC_000008.11 (11842524..11868137, complement)	APPS, CPSB
GNAI3	2773	Chromosome 1, NC_000001.11 (109548564..109595843)	RP5-1160K1.2, 87U6, ARCND1
ITPKB	3707	Chromosome 1, NC_000001.11 (226631690..226739327, complement)	IP3-3KB, IP3K, IP3K-B, IP3KB, PIG37
KIAA0101	9768	Chromosome 15, NC_000015.10 (64364994..64387687, complement)	L5, NS5ATP9, OEATC, OEATC-1, OEATC1, PAF, PAF15, p15(PAF), p15/PAF, p15PAF
PLOD2	5352	Chromosome 3, NC_000003.12 (146069439..146161495, complement)	LH2, TLH
VANGL1	81839	Chromosome 1, NC_000001.11 (115641953..115698224)	KITENIN, LPP2, STB2, STBM2

**Table 3:** Multivariate analysis of clinical data with/without seven-gene score for OS (n = 318).

Variables	Without seven-gene score		With seven-gene score	
HR	p (log-rank test)	HR	p (log-rank test)
Gender	1.33	0.195		
Age	1.04	0.0257	1.03	0.0496
Stages (Coef)	1.99	1.13 × 10^− 8^	2.03	5.95 × 10^− 8^
Cell types (Coef)	2.05	0.0261	1.58	0.1684
Seven-gene score (Coef)			2.61	1.91 × 10^− 10^

HR: hazard ratio; Coef: coefficient.
